# Conjugal DNA Transfer in Sodalis glossinidius, a Maternally Inherited Symbiont of Tsetse Flies

**DOI:** 10.1128/mSphere.00864-20

**Published:** 2020-11-04

**Authors:** Christopher G. Kendra, Chelsea M. Keller, Roberto E. Bruna, Mauricio H. Pontes

**Affiliations:** a Department of Pathology and Laboratory Medicine, Pennsylvania State University College of Medicine, Hershey, Pennsylvania, USA; b Department of Microbiology and Immunology, Pennsylvania State University College of Medicine, Hershey, Pennsylvania, USA; University of Wisconsin—Madison

**Keywords:** *Sodalis glossinidius*, insect endosymbiont, symbiont, transformation, conjugation, genetic modification, plasmid transfer, transposition, gene disruption, mutation, paratransgenesis, *Trypanosoma brucei*

## Abstract

Stable associations between insects and bacterial species are widespread in nature. This is the case for many economically important insects, such as tsetse flies. Tsetse flies are the vectors of Trypanosoma brucei, the etiological agent of African trypanosomiasis—a zoonotic disease that incurs a high socioeconomic cost in regions of endemicity. Populations of tsetse flies are often infected with the bacterium Sodalis glossinidius. Following infection, *S. glossinidius* establishes a chronic, stable association characterized by vertical (maternal) and horizontal (paternal) modes of transmission. Due to the stable nature of this association, *S. glossinidius* has been long sought as a means for the implementation of anti-*Trypanosoma* paratransgenesis in tsetse flies. However, the lack of tools for the genetic modification of *S. glossinidius* has hindered progress in this area. Here, we establish that *S. glossinidius* is amenable to DNA uptake by conjugation. We show that conjugation can be used as a DNA delivery method to conduct forward and reverse genetic experiments in this bacterium. This study serves as an important step in the development of genetic tools for *S. glossinidius*. The methods highlighted here should guide the implementation of genetics for the study of the tsetse-*Sodalis* association and the evaluation of *S. glossinidius-*based tsetse fly paratransgenesis strategies.

**IMPORTANCE** Tsetse flies are the insect vectors of T. brucei, the causative agent of African sleeping sickness—a zoonotic disease that inflicts a substantial economic cost on a broad region of sub-Saharan Africa. Notably, tsetse flies can be infected with the bacterium *S. glossinidius* to establish an asymptomatic chronic infection. This infection can be inherited by future generations of tsetse flies, allowing *S. glossinidius* to spread and persist within populations. To this effect, *S. glossinidius* has been considered a potential expression platform to create flies which reduce T. brucei stasis and lower overall parasite transmission to humans and animals. However, the efficient genetic manipulation of *S. glossinidius* has remained a technical challenge due to its complex growth requirements and uncharacterized physiology. Here, we exploit a natural mechanism of DNA transfer among bacteria and develop an efficient technique to genetically manipulate *S. glossinidius* for future studies in reducing trypanosome transmission.

## Abstract (precis)

Tsetse flies are the insect vectors of T. brucei, the causative agent of African sleeping sickness—a zoonotic disease that inflicts a substantial economic cost on a broad region of sub-Saharan Africa. Notably, tsetse flies can be infected with the bacterium *S. glossinidius* to establish an asymptomatic chronic infection. This infection can be inherited by future generations of tsetse flies, allowing *S. glossinidius* to spread and persist within populations. To this effect, *S. glossinidius* has been considered a potential expression platform to create flies which reduce T. brucei stasis and lower overall parasite transmission to humans and animals. However, the efficient genetic manipulation of *S. glossinidius* has remained a technical challenge due to its complex growth requirements and uncharacterized physiology. Here, we exploit a natural mechanism of DNA transfer among bacteria and develop an efficient technique to genetically manipulate *S. glossinidius* for future studies in reducing trypanosome transmission.

## INTRODUCTION

African trypanosomiasis or sleeping sickness is a zoonotic disease caused by the parasitic protozoan Trypanosoma brucei. Trypanosoma brucei is transmitted by tsetse flies (*Glossina* spp.; Diptera: *Glossinidae*), viviparous insects that feed exclusively on vertebrate blood (1, 2). In addition to T. brucei, natural populations of tsetse flies are often infected with strains of the Gram-negative bacterium Sodalis glossinidius (3–7). The establishment of *S. glossinidius* infection leads to a stable association, where the bacterium colonizes a number of tsetse fly tissues, including the salivary glands inhabited by T. brucei, without imposing a measurable burden on the flies (4, 8–12). Importantly, while *S. glossinidius* undergoes a predominantly maternal mode of transmission, being passed from mother to offspring during gestation (3, 8–12), this bacterium is also capable of paternal transmission during copulation (13), a phenomenon that may facilitate its colonization and spread within uninfected tsetse populations. Due to these particular characteristics, *S. glossinidius* has emerged as an attractive candidate for the implementation of tsetse fly paratransgenesis—a bioremediation strategy where bacteria capable of colonizing tsetse populations are used to express traits that inhibit *Trypanosoma* transmission (14–19).

The development of *Sodalis*-based paratransgenesis relies on the ability to genetically modify this bacterium. Although *S. glossinidius* has been isolated in axenic culture (4) and its genome has been sequenced (20), this bacterium has been proven refractory to artificial DNA transformation techniques. To date, two artificial transformation methods have been employed to introduce exogenous plasmid DNA into *S. glossinidius*: heat shock transformation and electroporation (8, 10, 13, 14, 16, 17, 21–26). While these transformation procedures were originally developed for Escherichia coli and popularized by the use of this organism as the workhorse of molecular biology (27, 28), they have proven to be both unreliable and inefficient as DNA delivery methods for *S. glossinidius*. Additionally, the use of natural DNA transfer methods such as conjugation has been hindered by the complex nutritional requirements and low growth rate of *S. glossinidius* (29), which undermine strategies to counterselect donor bacteria following DNA transfer.

**FIG 1:**
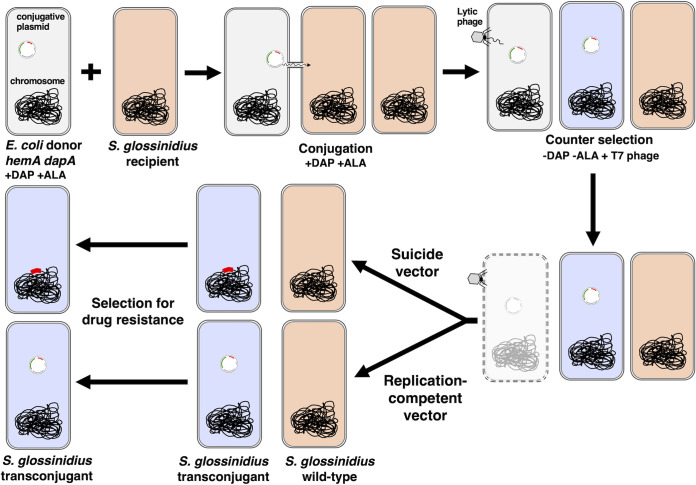
Representative workflow of the conjugation procedure developed for Sodalis glossinidius. Following the direction of the arrows, an E. coli *hemA dapA* donor strain (gray) is mixed with *S. glossinidius* (orange) and grown on medium containing DAP and ALA. Following the transfer of a conjugative plasmid (small circle) from the donor to the recipient cells, the mixture is exposed to E. coli-specific lytic bacteriophage T7. Following T7 absorption, cells are washed and plated on medium containing antibiotic but lacking DAP and ALA. The absence of these metabolic intermediates restricts growth of E. coli donor cells that escape killing by bacteriophage. Antibiotic selects for *S. glossinidius* transconjugants (blue). Suicide conjugative plasmids promote genetic modification (via transposition or homologous recombination) prior to being lost by segregation (top row). Replication-competent conjugative plasmids are maintained as autonomously replicating genetic elements (bottom row).

Here, we identify growth conditions enabling the counterselection of E. coli donor strains and demonstrate that *S. glossinidius* is amenable to uptake of DNA by conjugation (Fig. 1). We show that *S. glossinidius* exogenous DNA recipients (transconjugants) can be readily and reproducibly recovered after biparental mating with these E. coli donor strains. We use this technique for the implementation of forward genetic analysis through the generation of random transpositions with a Himar1 Mariner and mini-Tn*5* transposition systems and of reverse genetics by insertionally inactivating a number of chromosomal genes using suicide vectors. This work establishes conjugation as a reliable DNA delivery method for the genetic manipulation of *S. glossinidius* and will greatly facilitate the study of this bacterium and the evaluation of methods for tsetse paratransgenesis.

## RESULTS

### Prolonged incubation of Escherichia coli *dapA hemA* donor strain on rich medium lacking diaminopimelic acid and δ-aminolevulinic acid gives rise to suppressor mutants that no longer require these nutrients.

During conjugation, donor and recipient cells must come into close physical proximity to enable DNA transfer through a pilus (30). Subsequently, recipient cells which have received DNA (transconjugants) are recovered on plates containing solid medium that restricts the growth of donor and recipient cells that did not receive the desired DNA molecule. Transconjugants are positively selected based on the presence of a genetic marker within the transferred DNA (i.e., antibiotic-resistant gene). However, because donor cells retain a copy of the transferred DNA, they have to be eliminated by other means. Classically, this is achieved through nutrition-based auxotrophy counterselections, where the growth of the donor is hindered by plating the conjugation mixture on defined medium lacking a nutrient synthesized by the recipient, but not the donor (e.g., a particular amino acid). However, amino acid-auxotrophy-based strategies cannot be used to counterselect donor cells in conjugation mixtures with *S. glossinidius*. This is because *S. glossinidius* is a slow-growing microaerophilic bacterium with complex nutritional requirements (4, 29), and defined solid medium recipes that support the formation of colonies are currently unavailable. To overcome this hurdle, we sought to exploit well-characterized E. coli donor strains containing alternative auxotrophies that can be used for counterselection on complex media.

**FIG 2:**
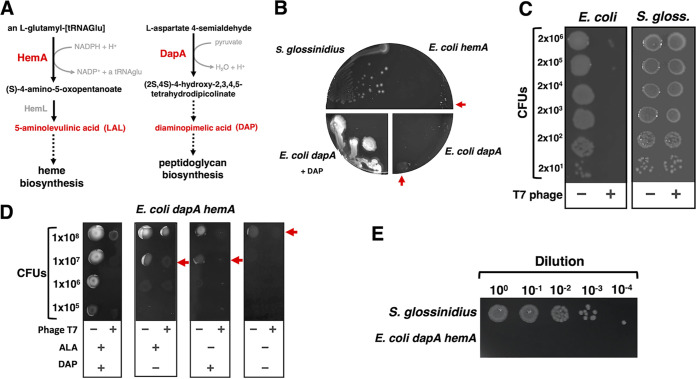
Counterselection of E. coli *hemA dapA* donor on BHIB agar. (A) Schematics depicting the reactions catalyzed by HemA (left side) and DapA (right side), enzymes required for the biosynthesis of heme and peptidoglycan, respectively. Complementation of growth medium with the metabolic intermediates depicted in red is used to support the growth of *hemA* and *dapA* mutants. (B) Growth of wild-type *S. glossinidius*, E. coli *hemA* (MP1182), and E. coli *dapA* (BW29427) on BHIB agar lacking or containing DAP. Plates were photographed after 8 days of incubation at 27°C under microaerophilic conditions. Red arrows indicate residual growth. (C) Growth of wild-type E. coli (MG1655) and *S. glossinidius* following 120 min of incubation, at room temperature, in 10 mM MgCl_2_ or 10 mM MgCl_2_ containing phage T7. Cell suspensions were diluted, and 5 μl was spotted on plates. Escherichia coli was incubated at 37°C on LB for 16 h. Sodalis glossinidius was incubated under microaerophilic conditions at 27°C on BHIB for 8 days. (D) Growth of E. coli *dapA hemA* (MP1554) on BHIB agar with various combinations of ALA and DAP. Cells were incubated at room temperature for 120 min in 10 mM MgCl_2_ or 10 mM MgCl_2_ containing phage T7. Cultures were diluted, and 5 μl was plated on BHIB agar. Plates were incubated at 37°C for 16 h. Red arrows indicate residual growth. (E) Growth of *S. glossinidius* and E. coli *dapA hemA* (MP1554) on BHIB agar lacking ALA and DAP. Bacteria were grown separately on plates in a mock conjugation experiment, subsequently exposed to phage T7, washed, diluted, and spotted on BHIB as described for panel C. Plates were incubated for 8 days at 27°C under microaerophilic conditions. Images depict representative plates of at least 3 independent experiments.

In E. coli, the *dapA* gene encodes a 4-hydroxy-tetrahydrodipicolinate synthase and the *hemA* gene encodes a glutamyl-tRNA reductase. These enzymes are required for the biosynthesis of peptidoglycan and heme, respectively. While mutations in *dapA* give rise to a requirement for diaminopimelic acid (DAP), mutations in *hemA* create a requirement for δ-aminolevulinic acid (ALA) or heme, respectively (Fig. 2A) (31, 32). As DAP and ALA are usually not present in complex microbial medium components, E. coli donor strains containing these mutations are often used to select transconjugants on rich medium such as Luria-Bertani (LB) (33, 34). Sodalis glossinidius forms colonies 5 to 10 days following plating on rich media, such as brain heart infusion-blood (BHIB) agar. Therefore, we sought to determine if E. coli donor strains containing mutations in *dapA* and/or *hemA* were able to grow on BHIB agar. Escherichia coli *dapA* and *hemA* strains were streaked on BHIB agar along with *S. glossinidius* and incubated for 8 days under microaerophilic conditions. Following incubation, *S. glossinidius* formed small colonies as expected (Fig. 2B). In contrast, *dapA* and *hemA* strains displayed residual growth at the inoculation sites on the plates (Fig. 2B). Control BHIB plates supplemented with DAP supported the growth of the E. coli *dapA* strain, which formed large colonies following 8 days of incubation (Fig. 2B).

The abovementioned results suggested that it might be possible to counterselect a *dapA* or *hemA* E. coli donor strain on BHIB agar following conjugation with *S. glossinidius*. We therefore attempted to recover *S. glossinidius* transconjugants under a number of mating conditions. We found that E. coli *dapA* suppressor mutants that are able to grow in the absence of DAP emerge at high frequency following 5 or 16 h of mating, where strains are mixed at ratios of 50 *S. glossinidius* bacteria to 1 E. coli bacterium or 2,500 *S. glossinidius* bacteria to 1 E. coli bacterium, respectively (see Fig. S1A in the supplemental material). Indeed, even in an E. coli donor strain containing both *dapA* and *hemA* mutations, suppressor mutants that do not require DAP and ALA emerged at a relatively high frequency following 8 days of incubation on BHIB agar (approximately 2 × 10^−7^ CFU) (Fig. S1B). Together, these results indicate that the introduction of *dapA* and *hemA* mutations in an E. coli donor strain can be used as part of a counterselection strategy but is not sufficient to retrieve *S. glossinidius* transconjugants.

10.1128/mSphere.00864-20.1 FIG S1 Growth of E. coli *dapA* and *dapA hemA* strains on BHIB agar. (A) A representative plate of an E. coli *dapA* and *S. glossinidius* conjugation mixture grown for 8 days, at 27°C on BHIB agar under microaerophilic conditions. The square highlights a magnified portion of the plate depicting small *S. glossinidius* colonies and large mucoid E. coli *dapA* suppressor colonies that are able to grow in the absence of DAP (A. I. Bukhari and A. L. Taylor, J Bacteriol 105:844–854, 1971, https://doi.org/10.1128/JB.105.3.844-854.1971). (B) Representative BHIB agar plates seeded with 2 × 10^9^ CFU of E. coli *dapA hemA.* Where indicated, plates were supplemented with DAP or ALA, or cells were pretreated with bacteriophage T7 lysate. Plates were incubated for 7 days, at 27°C and under microaerophilic conditions. Plates displaying confluent growth were imaged following 2 days of incubation, when growth became apparent. Download FIG S1, TIF file, 2.4 MB. Copyright © 2020 Kendra et al. 2020 Kendra et al. This content is distributed under the terms of the Creative Commons Attribution 4.0 International license.

### *dapA hemA* donor suppressors can be eliminated using an E. coli-specific lytic bacteriophage.

T7 is a lytic bacteriophage (phage) with a narrow host range. This phage typically infects certain E. coli and closely related *Shigella* strains, as well as certain *Yersinia* strains (35). Given the specificity of T7, we wondered if we could use this phage to target E. coli cells in conjugation mixtures with *S. glossinidius*. Therefore, we sought to determine if *S. glossinidius* was immune to killing by phage T7. We established that despite causing a decrease of 5 orders of magnitude in the number of CFU in cultures of the E. coli donor strain, exposure to phage T7 did not decrease CFU counts in *S. glossinidius* cultures, indicating that the latter bacterium is immune to T7 killing (Fig. 2C).

Given these results, we decided to examine the effect of phage T7 on the emergence of E. coli *dapA hemA* suppressors that can grow in the absence of DAP, ALA, or both. We plated the E. coli *dapA hemA* donor on BHIB agar in the presence or absence of DAP, ALA, and/or phage T7. Consistent with previous results (Fig. 2B and Fig. S1B), removal of either or both DAP and ALA caused a decrease in cell survival of the E. coli donor (Fig. 2D). The presence of phage T7 alone also decreased the survival of the E. coli donor under all conditions tested (Fig. 2D). In the absence of DAP and ALA, phage T7 lowered the number of donor cells by over 9 orders of magnitude, effectively preventing the emergence of E. coli *dapA hemA* suppressors that can grow in the absence of DAP and ALA (Fig. 2D and Fig. S1B). Importantly, after population expansion of the E. coli donor for 16 h in a mock conjugation experiment, exposure to phage T7 was sufficient to prevent the emergence of E. coli *dapA hemA* suppressors within the time window permitting *S. glossinidius* to form colonies (i.e., 8 days [Fig. 2E]). Together, these results suggested that *S. glossinidius* cells can be isolated from conjugation mixtures with an E. coli *dapA hemA* donor following exposure to T7 phage.

### Conjugation of transposition systems for random mutagenesis of *S. glossinidius*.

**FIG 3:**
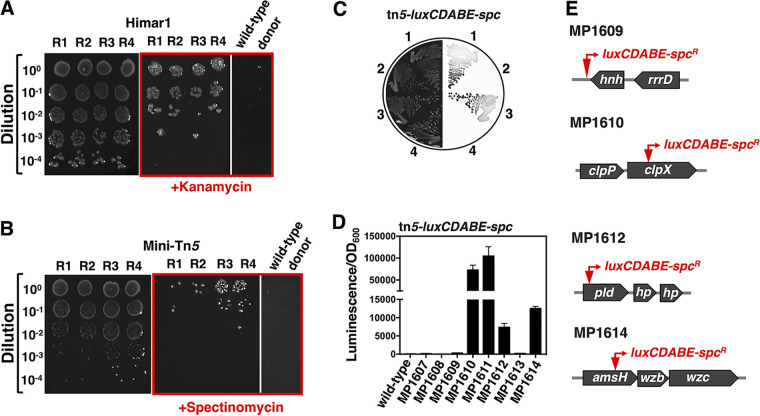
Transposition mutagenesis in *S. glossinidius*. (A) Serial dilutions of conjugation mixtures of *S. glossinidius* and E. coli *dapA hemA* (MP1554) harboring the suicide vector encoding a Mariner transposon system (Himar1), pMarC9-R6k. Five microliters of cell suspension was spotted on BHIB agar (left panel) or BHIB agar supplemented with kanamycin (middle panel). Individually grown conjugation partners, *S. glossinidius* and E. coli *dapA hemA* (MP1554) pMarC9-R6k, were also spotted on BHIB agar supplemented with kanamycin (right panel). The red box indicates plates containing kanamycin. Note that dots at the donor lane correspond to locations where pipette tips punctured the agar. Similar dots are present in lane R2 and on some lanes of panel B. (B) Serial dilutions of conjugation mixtures of *S. glossinidius* and E. coli *dapA hemA* (MP1554) harboring the suicide vector encoding the Tn*5*-based promoter-probe transposition system, pUTmini-Tn*5*-*luxCDABE*-Spc. Five microliters of cell suspension was spotted on BHIB agar (left panel) or BHIB agar supplemented with spectinomycin (middle panel). Individually grown *S. glossinidius* and E. coli *dapA hemA* (MP1554) pUTmini-Tn*5*-*luxCDABE*-Spc were spotted on BHIB agar supplemented with spectinomycin (right panel). Red box indicates plates containing spectinomycin. (C) Transconjugants obtained in a conjugation experiment described for panel B were purified on BHIB agar supplemented with spectinomycin. Luminescence signals of four distinct clones are depicted on the right side of the figure. Plates were incubated for 8 days at 27°C under microaerophilic conditions. Images depict representative plates of at least 3 independent experiments. (D) Quantification of luminescence signals derived from selected *S. glossinidius* mini-Tn*5*-*luxCDABE-spc^R^* transconjugants obtained as described for panel B. Error bars represent standard deviations from three technical replicates. (E) Schematic illustration depicting locations of mini-Tn*5*-*luxCDABE-spc^R^* transposition insertions in selected *S. glossinidius* clones—*hnh* (SGGMMB4_03814), *clpX* (SGGMMB4_01523), *pld* (SGGMMB4_05728), and *amsH* (SGGMMB4_02193).

Transposable elements have played a pivotal role in the development of forward genetics studies in bacterial species (36, 37) and have been previously used in studies of *S. glossinidius* (22). We therefore attempted to use conjugation for the delivery of stable transposition systems encoded within mobilizable suicide vectors into this bacterium. Following conjugation, *S. glossinidius* transconjugants were readily recovered by selecting for the antibiotic markers encoded within each transposon (Fig. 3A and B).

A number of controls indicated that these *S. glossinidius* cells were true transconjugants resulting from random transposition events, originating from the mobilized suicide vector. First, no antibiotic-resistant clones were recovered from *S. glossinidius* cells which were not conjugated with the E. coli donor. Hence, the emergence of antibiotic resistance was linked to a physical interaction with the donor strain (Fig. 3A and B). Second, antibiotic-resistant clones of *S. glossinidius* remained sensitive to ampicillin, indicating that they did not retain the suicide vector, either as an autonomous replicating episome or as a vector integrated into the chromosome (Table 1). Third, conjugation experiments involving promoter-probe transposition systems, such as Tn*5*-*luxCDABE*-*Spc* (38), yielded a population of antibiotic-resistant *S. glossinidius* clones displaying heterogeneous reporter-gene expression (Fig. 3C and D). Thus, the recovered transconjugant clones emerged from distinct transposition events (Fig. 3C and D). Consistent with these observations, mapping putative transposition events in some of these clones revealed transposon insertions into distinct chromosomal locations (Fig. 3E). Importantly, while the conjugation efficiency varied with particular transposition systems, transconjugants were reproducibly recovered at high frequency (1.40 × 10^−3^ to 1.84 × 10^−2^) (Table 1 and Table S1). Together, these results demonstrate that conjugation can be reliably used to deliver transposition systems into *S. glossinidius*.

**TABLE 1:** Summary of conjugation experiments^*a*^

Plasmid	Origin of replication	Positive selection		Conjugation efficiency	Plasmid retention (Amp^r^)
		Transposition or insertion	Plasmid		
pFAJ1815	R6K γ	Kanamycin	Ampicillin	1.80E−03 (N = 14)	0/24
pUT-miniTn*5*-lux-Km2	R6K γ	Kanamycin	Ampicillin	6.82E−03 (N = 21)	0/24
pUT-Tn*5*-GFP	R6K γ	Kanamycin	Ampicillin	5.56E−03 (N = 12)	N/D
pMarC9-R6K	R6K γ	Kanamycin	Ampicillin	1.84E−02 (N = 5)	N/D
pUT-miniTn*5*-lux-Sp	R6K γ	Spectinomycin	Ampicillin	1.40E−03 (N = 8)	0/21

a N, number of experiments; Amp^r^, ampicillin resistance; N/D, not determined.

10.1128/mSphere.00864-20.3 TABLE S1 Raw mating experiments. Download Table S1, XLSX file, 0.01 MB. Copyright © 2020 Kendra et al. 2020 Kendra et al. This content is distributed under the terms of the Creative Commons Attribution 4.0 International license.

### Conjugation of suicide vectors for targeted gene disruption in *S. glossinidius*.

**FIG 4:**
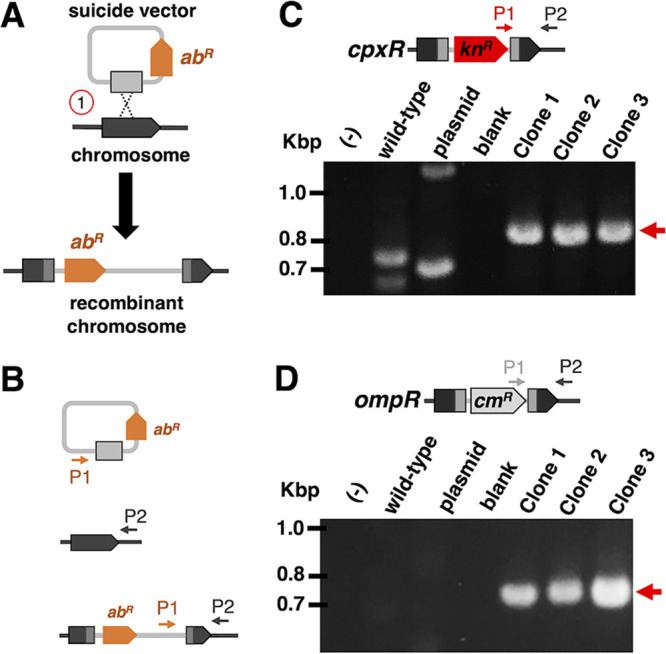
Gene targeting in *S. glossinidius* by insertional inactivation. (A) General schematic depicting the integration of a suicide vector harboring an antibiotic-resistant marker (*ab^R^*, orange) into a specific chromosomal region. A homologous recombination event (1) between homologous fragments on the chromosome (dark gray) and plasmid (light gray) is shown. (B) General schematic cartoons depicting the annealing locations of the confirmatory primers (P1 and P2) on the targeted chromosome location and suicide vector. (C and D) Agarose gel images of electrophoresed PCR products obtained with primers P1 and P2. Lanes indicate DNA templates used in each PCR: negative control [(−)], wild-type *S. glossinidius* genomic DNA (wild-type), suicide plasmid vector (plasmid), and transconjugant *S. glossinidius* clones (clone 1, clone 2, and clone 3). Bands corresponding to PCR products spanning an insertion point of the suicide plasmid are indicated with red arrows. In panel C, note the presence of unspecific bands on the lanes corresponding to *S. glossinidius* wild-type and plasmid DNA. The identity of PCR bands has been verified by DNA sequencing.

We tested whether we could use conjugation for the delivery of replication-deficient suicide plasmids designed for targeted gene disruption. In contrast to transposition, this reverse genetic strategy relies on homologous recombination functions encoded by the host bacterium (39). In its simplest form, insertional disruptions can be generated through single homologous recombination events between the target gene and a homologous fragment cloned in a suicide vector—i.e., a Campbell-like integration (Fig. 4A). We employed this strategy to target the transcriptional regulators encoded by *S. glossinidius cpxR* and *ompR* homologs. That is, following conjugation, we were able to recover kanamycin- and chloramphenicol-resistant *S. glossinidius* clones which, upon PCR analyses, were shown to harbor plasmid insertions in the expected chromosomal locations (Fig. 4B and C). Taken together, these results demonstrate that conjugation can be used for the delivery of suicide vectors for targeted gene disruption in *S. glossinidius*.

## DISCUSSION

In the current study, we established conditions permitting the counterselection of E. coli on BHIB agar. We use these conditions to hinder the growth of E. coli DNA donor strains following mating and demonstrate that the slow-growing, fastidious bacterium *S. glossinidius* is receptive to DNA transfer by conjugation. We employed conjugation to perform random transposition and targeted mutagenesis in *S. glossinidius*, effectively implementing efficient methods to carry out forward and reverse genetics.

The incorporation of exogenous DNA in bacterial cells occurs in a two-step process. First, DNA must cross the cellular membrane, reaching the cytoplasm. Second, DNA must become established within the recipient cell, being stably transmitted to subsequent generations. In *S. glossinidius*, these two steps have been previously achieved through heat shock transformation and electroporation (8, 10, 13, 14, 16, 17, 21–26). However, these artificial transformation methods have been proven inefficient and unreliable for the introduction of exogenous DNA into this bacterium. Not surprisingly, genetic manipulations of *S. glossinidius* have been largely restricted to the introduction of autonomously replicating circular plasmids—usually employed for the expression of foreign genes in this bacterium—because uptake and establishment of such DNA molecules are frequent and stable enough to enable their sporadic detection following artificial transformation (8, 10, 13, 14, 16, 17, 23–26).

Resistance to artificial transformation methods is not uncommon among bacterial species. For instance, Salmonella enterica is resistant to DNA transformation by heat shock. Interestingly, mutations that alter the chemical composition of the lipopolysaccharide (LPS) can increase heat shock transformation efficiency in this bacterium by 2 orders of magnitude (40). Likewise, Klebsiella pneumoniae is recalcitrant to transformation by electroporation. Treatment of cells with the chelating agent EDTA increases transformation by 4 orders of magnitude, presumably because this compound helps to permeabilize *Klebsiella* cells during electric pulse by removing excess capsular exopolysaccharide and/or destabilizing the LPS (41). Similar to *Klebsiella*, species belonging to the *Bacteroides* genus are also resistant to DNA transformation by electroporation. For example, in Bacteroides fragilis, the uptake of electroporated DNA is so rare that populations of transformed cells must be expanded for 12 h prior to isolation of transformants on selective agar plates (42). Fortunately, *Bacteroides* species both are susceptible to DNA transfer by natural methods such as conjugation and can be propagated under conditions that easily restrict the growth of donor bacteria (43–46).

In contrast, *S. glossinidius* is a fastidious, slow-growing species with a poorly characterized physiology. Solid medium formulations that support the growth of *S. glossinidius* and hinder the growth of commonly used DNA donor strains are currently lacking. Because such strains quickly overtake *S. glossinidius* during growth on solid medium (Fig. 2B) (29), its propensity to take up DNA via conjugation had not been previously tested. To circumvent this problem, we implemented a strategy whereby growth of donor bacteria could be hindered by three distinct counterselections. Specifically, we introduced a *hemA* mutation into a commonly used E. coli *dapA* DNA donor, producing a strain with metabolic requirements for both DAP and ALA—two compounds not found in complex medium formulations that support the growth of *S. glossinidius*. Following mating with *S. glossinidius*, this E. coli strain is selectively killed by a lytic bacteriophage, and growth of surviving donor cells is suppressed by plating the conjugation mixture on selective plates lacking DAP and ALA.

Whereas this strategy allowed us to demonstrate that conjugation can be used to efficiently transfer DNA to *S. glossinidius* (Fig. 3 and 4; Table 1), other strategies may be employed to hinder growth or eliminate DNA donor cells. These include the conditional expression of toxic genes (47–53) and/or the exploitation of metabolic pathways present in donor cells to generate toxic metabolic intermediates that inhibit their growth (54, 55). Notably, a recent study has reported the development of a defined liquid medium formulation for the growth of *S. glossinidius* (56). While in theory this defined medium could be used as part of a counterselection strategy aimed at eliminating DNA donor cells (i.e., the implementation of amino acid auxotrophies), we determined that this formulation does not support the formation of *S. glossinidius* colonies (see Fig. S2 in the supplemental material).

10.1128/mSphere.00864-20.2 FIG S2 Growth of *S. glossinidius* on various agar plates. (A) Representative plate of a BHI agar plate of *S. glossinidius* growth for 13 days, at 27°C under microaerophilic conditions. The bottom square highlights a magnified portion of the plate depicting relatively large, uniformly sized colonies. (B) Representative plate of an LB glucose agar plate of *S. glossinidius* growth for 13 days, at 27°C under microaerophilic conditions. The bottom square highlights a magnified portion of the plate depicting relatively small colonies and rare, larger colonies. (C) Representative plate of an SGM11 medium (R. J. Hall, L. A. Flanagan, M. J. Bottery, V. Springthorpe, et al., mBio 10:e02106-18, 2019, https://doi.org/10.1128/mBio.02106-18) agar plate of *S. glossinidius* growth for 25 days, at 27°C under microaerophilic conditions. The bottom square highlights a magnified portion of the plate. No growth is observed. Download FIG S2, TIF file, 2.0 MB. Copyright © 2020 Kendra et al. 2020 Kendra et al. This content is distributed under the terms of the Creative Commons Attribution 4.0 International license.

The basic genetic modifications performed in this study serve as a proof of principle and should greatly facilitate the development and implementation of *S. glossinidius*-based paratransgenesis in tsetse flies. While we exploit conjugation for the transfer of suicide vectors designed for targeted and random mutagenesis into *S. glossinidius*, this process can be used for the delivery of any mobilizable genetic element harboring an origin of transfer (*oriT*) to this bacterium (Fig. 1). These include replication-competent plasmids or other episomes encoding an array of functions, such as targeted transposition (57) and advanced genome editing CRISPR-based systems (58). From a broader perspective, this study highlights the application of conjugation as a reliable DNA delivery mechanism for *S. glossinidius* and potentially other fastidious bacterial species with undeveloped genetic methods. These include many cultured insect-associated bacteria such as “*Candidatus* Arsenophonus arthropodicus,” “*Candidatus* Arsenophonus triatominarum,” “*Candidatus* Sodalis melophagi,” Hamiltonella defensa, Serratia symbiotica, and Spiroplasma poulsonii (59–64).

## MATERIALS AND METHODS

### Microbial strains, phages, plasmids, and growth conditions.

Microbial strains, phages, and plasmids used in this study are presented in Table S2 in the supplemental material. Unless indicated, all E. coli strains were propagated at 37°C or 30°C in LB broth or agar (1.5% [wt/vol]). Sodalis glossinidius was grown at 27°C in brain heart infusion broth supplemented with 10 mM MgCl_2_ (BHI). Growth of *S. glossinidius* on agar (1.2% [wt/vol]) plates of BHI, BHI supplemented with 10% defibrinated horse blood (BHIB), LB supplemented with 30 mM glucose, or SGM11 (56) was carried out under microaerophilic conditions, which were achieved using either BD GasPak EZ Campy gas-generating sachets or a gas mixture (5% oxygen-95% CO_2_). For both E. coli and *S. glossinidius*, growth in liquid medium was carried out with aeration (250 rpm). Sodalis glossinidius liquid cultures were typically propagated in 500-ml orange-cap medium storage bottles containing 150 to 250 ml of medium broth. When required, medium was supplemented with ampicillin (100 μg/ml), chloramphenicol (20 μg/ml for E. coli and 10 μg/ml for *S. glossinidius*), kanamycin (50 μg/ml for E. coli and 25 μg/ml for *S. glossinidius*), spectinomycin (100 μg/ml for E. coli and 30 μg/ml for *S. glossinidius*), δ-aminolevulinic acid (ALA, 100 μg/ml), or diaminopimelic acid (DAP, 60 μg/ml). Anhydrotetracycline was used at 0.2 μg/ml.

10.1128/mSphere.00864-20.4 TABLE S2 Microbial strains, phages, and plasmids used in this study. Download Table S2, XLSX file, 0.01 MB. Copyright © 2020 Kendra et al. 2020 Kendra et al. This content is distributed under the terms of the Creative Commons Attribution 4.0 International license.

### Construction of suicide vectors for targeted gene disruption.

Oligonucleotide sequences used in this study are presented in Table S3. Phusion high-fidelity DNA polymerase (New England BioLabs) was used in PCR with *S. glossinidius* genomic DNA or plasmid pKD3 or pKD4 (65). While *S. glossinidius* genomic DNA was used for the amplification of fragments within targeted genes (*cpxR* and *ompA*), plasmids were used for the amplification of chloramphenicol- and kanamycin-resistant genes. PCR fragments were assembled in the backbone of suicide vector pAOJ15 (52), previously digested with BamHI and EcoRI, using NEBuilder HiFi DNA assembly (New England BioLabs). Assembly reaction mixtures were transformed into E. coli cells by heat shock (27), and transformants were selected on LB plates containing either ampicillin and chloramphenicol or kanamycin. The integrity of constructs was verified by PCR using primers flanking the ligation points between different fragments within each plasmid. PCR products were also purified from agarose gels using the Monarch DNA gel extraction kit (New England BioLabs) and subjected to DNA sequencing.

10.1128/mSphere.00864-20.5 TABLE S3 Oligonucleotide sequences used in this study. Download Table S3, XLSX file, 0.01 MB. Copyright © 2020 Kendra et al. 2020 Kendra et al. This content is distributed under the terms of the Creative Commons Attribution 4.0 International license.

### Construction of *hemA* Escherichia coli strains.

Oligonucleotide sequences used in this study are presented in Table S3. Escherichia coli BW29427 (also known as WM3064) or S17-1 harboring plasmid pSIM6 (66) was grown overnight in LB medium supplemented with 100 μg/ml of ampicillin and 60 μg/ml of DAP at 30°C and 250 rpm. Cultures were diluted (1:100) in 30 ml of the same medium and grown for approximately 2.5 h (optical density at 600 nm [OD_600_], ∼0.35 to 0.4). The cultures were then grown in a water bath for 30 min at 42°C and 250 rpm (final OD_600_ of ∼0.6 to 0.8). Cultures were immediately transferred to a 50-ml conical tube and collected by centrifugation at 7,000 rpm using an F-35-6-30 5430/5430R rotor (Eppendorf). Cultures were centrifuged at 4°C, 7,000 rpm, for 2.5 min, and cells were resuspended in 40 ml of ice-cold distilled water (dH_2_O). Cells were collected again by centrifugation, and this washing procedure was repeated a second time. Finally, cells were resuspended in 150 μl of ice-cold dH_2_O. Homologous recombination was obtained by electroporating 70 μl of cell suspension with 10 μl of the purified PCR product, generated with primers 212 and 213 and plasmid pKD3 as the template (65). Recombinants were recovered on LB plates supplemented with 20 μg/ml of chloramphenicol, 60 μg/ml of DAP, and 100 μg/ml of ALA. Chloramphenicol clones were screened for the inability to grow on LB in the absence of ALA.

### Transposition mapping.

Mapping of mini-Tn*5*-*luxCDABE-spc^R^* (38) transposition insertions was carried out as described previously (67).

### Preparation of bacteriophage T7 solutions.

Escherichia coli MG1655 cultures were grown overnight in LB broth at 37°C and 250 rpm. One milliliter of overnight cultures was used to inoculate 100 ml of fresh LB broth. Cultures were allowed to grow for 2 h at 37°C and 250 rpm to an OD_600_ of ∼0.3 to 0.4 and then infected with 200 μl bacteriophage T7 stock lysate. After 3 to 4 h of lysis, cells were transferred to conical tubes and a 1/1,000 volume of chloroform was added to each tube. Tubes were vortexed for 1 min, and cell debris was pelleted by centrifugation at 7,000 rpm using an F-35-6-30 5430/5430R rotor (Eppendorf). Debris was centrifuged for 2.5 min at room temperature, and the supernatant was passed through an 0.22-μm polyether sulfone membrane filter. Supernatants containing bacteriophage T7 were concentrated using an Amico Ultra-15 centrifugal filter (Millipore), and LB broth was replaced with 1 to 4 ml of solution of 10 mM MgCl_2_. Lysates were subsequently sterilized by filtration through an 0.22-μm polyether sulfone membrane and stored at 4°C.

### Bacteriophage T7 killing assay.

Escherichia coli MG1655 and *S. glossinidius* cells were collected by centrifugation and resuspended, at concentrations of 1 × 10^8^ to 1 × 10^9^ CFU, in 1 ml of 10 mM MgCl_2_ or 10 mM MgCl_2_ containing phage T7. Cells were incubated on benches at room temperature for 120 min. Cell suspensions were then diluted, and 5 μl was spotted on agar plates. Escherichia coli was incubated at 37°C on LB for 16 h. Sodalis glossinidius was incubated under microaerophilic conditions at 27°C on BHIB for 8 days.

### Conjugation conditions.

Escherichia coli donor strains were grown overnight (18 h) in LB broth supplemented with 100 μg/ml of ampicillin, 60 μg/ml of DAP, and/or 100 μg/ml ALA, at 37°C and 250 rpm. Cultures were diluted 1:100 into 3 ml of fresh medium lacking ampicillin and grown for 2 h, to an optical density (OD_600_) of ∼0.15 to 0.30. Sodalis glossinidius was grown in 150 to 250 ml of BHI broth to an OD_600_ of ∼0.4 to 1.0. Cells were then collected by centrifugation at 7,000 rpms using an F-35-6-30 5430/5430R rotor (Eppendorf). Cells were centrifuged for 5 min at room temperature and subsequently resuspended in BHI broth to a final OD_600_ of ∼10 to 20. Escherichia coli donor and *S. glossinidius* recipient cells were mixed (20 μl donor solution to 200 μl recipient solution), and 50-μl aliquots were spotted on an 0.45-μm filter paper resting on BHIB agar supplemented with DAP and/or ALA. Control matings, containing only donor or recipient cells, were set up alongside, and plates were incubated for 12 to 16 h at 27°C under microaerophilic conditions. Cells on filters were resuspended in a solution of bacteriophage T7 and incubated for 2 h at room temperature. The cell mixture was collected by centrifugation (5 min at 7,000 rpm at room temperature using an F-35-6-30 5430/5430R Eppendorf rotor) and washed three times with phosphate-buffered saline (PBS) to remove residual DAP and ALA. Cell pellets were then resuspended in T7 solution, diluted, and plated on BHIB agar with the appropriate antibiotics and 0.2 μg/ml of anhydrotetracycline.

### Quantification of bioluminescence in bacterial cultures.

Light production by individual clones grown in BHI broth was measured using a SpectraMax i3x plate reader (Molecular Devices). Luminescence signals were normalized by optical densities of the cultures (absorbance at 600 nm).

### Image acquisition, analysis, and manipulation.

DNA separated by agarose gel electrophoresis and light production by *S. glossinidius* colonies were detected using an Amersham Imager 680 (GE Healthcare). When oversaturated, the intensity of signals in images was adjusted across the entire image using Preview (Apple).
