# Supplementary figures and images for: Conjugal DNA Transfer in Sodalis glossinidius, a Maternally Inherited Symbiont of Tsetse Flies
Source: mSphere. 2020 Nov 4;5(6):e00864-20. doi: 10.1128/mSphere.00864-20 (PMC7643829; doi:10.1128/mSphere.00864-20)

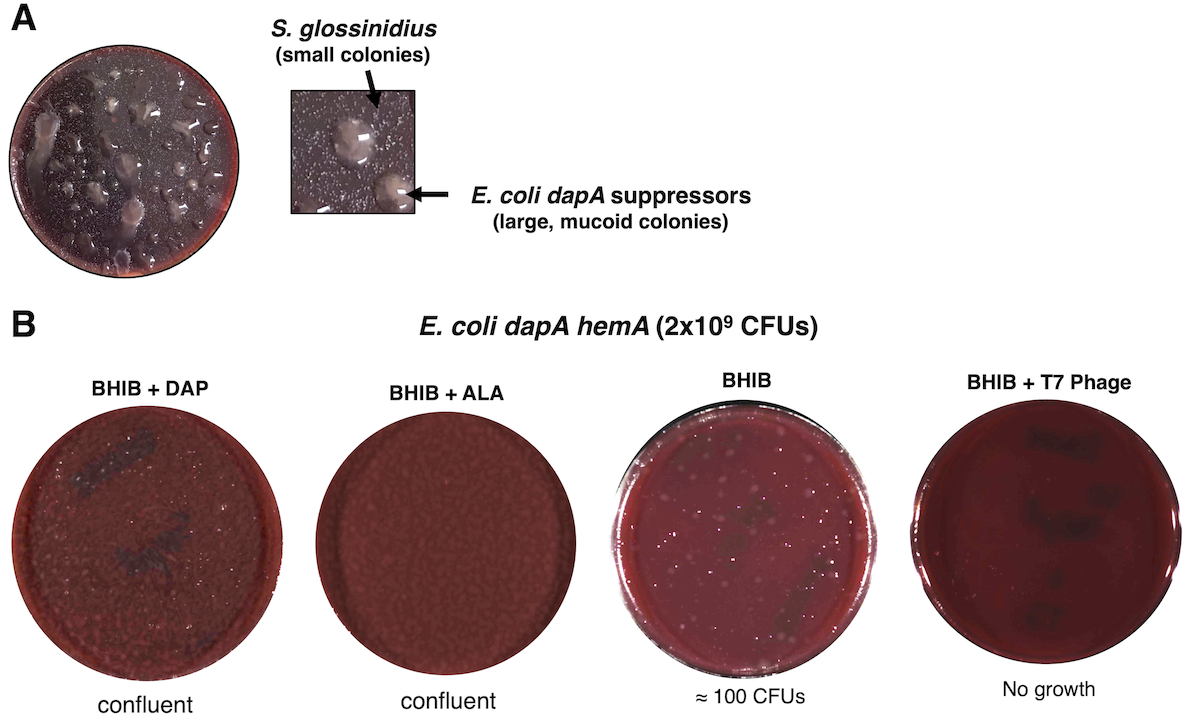

Supplement: FIG S1 [file mSphere.00864-20-sf001.tif]

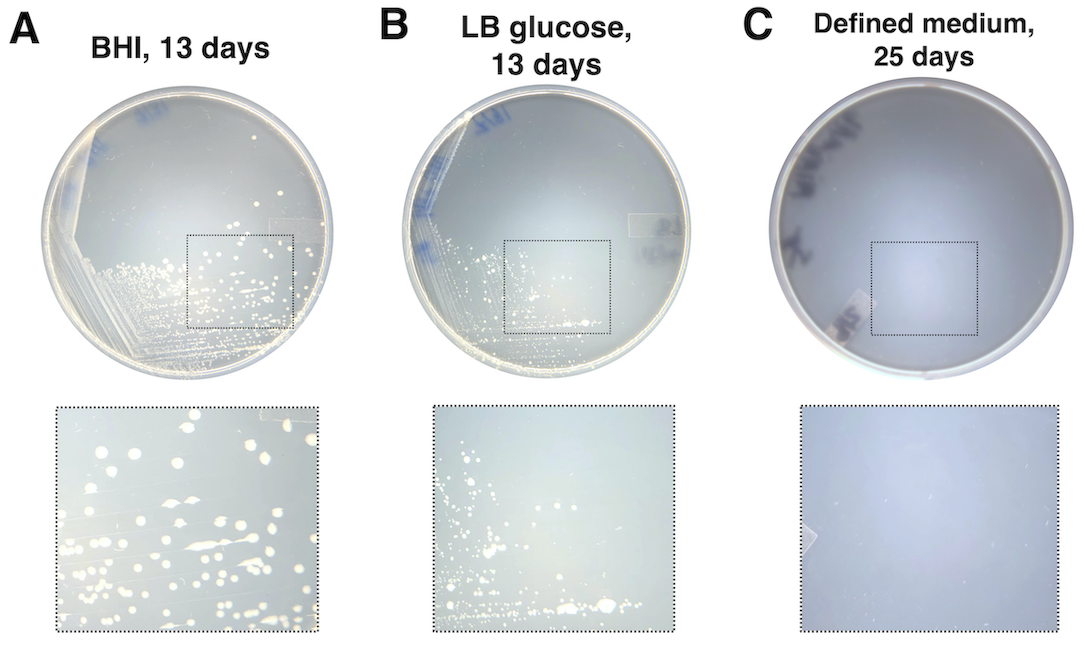

Supplement: FIG S2 [file mSphere.00864-20-sf002.tif]
